# A confocal microscopic study of solitary pulmonary neuroendocrine cells in human airway epithelium

**DOI:** 10.1186/1465-9921-6-115

**Published:** 2005-10-10

**Authors:** Markus Weichselbaum, Malcolm P Sparrow, Elisha J Hamilton, Philip J Thompson, Darryl A Knight

**Affiliations:** 1 Asthma and Allergy Research Institute, Sir Charles Gairdner Hospital, Nedlands, 6009, Western Australia; 2 Centre for Asthma, Allergy and Respiratory Research, University of Western Australia, 6009; 3 Department of Physiology, University of Western Australia, Nedlands, 6009, Western Australia; 4 Heart Research Institute, Royal North Shore Hospital, The University of Sydney NSW 2006 Australia; 5 James Hogg iCAPTURE center for Cardiovascular and Respiratory Research, St. Pauls Hospital, University of British Columbia, Vancouver, BC V6Z 1Y6, Canada

## Abstract

**Background:**

Pulmonary neuroendocrine cells (PNEC) are specialized epithelial cells that are thought to play important roles in lung development and airway function. PNEC occur either singly or in clusters called neuroepithelial bodies. Our aim was to characterize the three dimensional morphology of PNEC, their distribution, and their relationship to the epithelial nerves in whole mounts of adult human bronchi using confocal microscopy.

**Methods:**

Bronchi were resected from non-diseased portions of a lobe of human lung obtained from 8 thoracotomy patients (Table 1) undergoing surgery for the removal of lung tumors. Whole mounts were stained with antibodies to reveal all nerves (PGP 9.5), sensory nerves (calcitonin gene related peptide, CGRP), and PNEC (PGP 9.5, CGRP and gastrin releasing peptide, GRP). The analysis and rendition of the resulting three-dimensional data sets, including side-projections, was performed using NIH-Image software. Images were colorized and super-imposed using Adobe Photoshop.

**Results:**

PNEC were abundant but not homogenously distributed within the epithelium, with densities ranging from 65/mm^2 ^to denser patches of 250/mm^2^, depending on the individual wholemount. Rotation of 3-D images revealed a complex morphology; flask-like with the cell body near the basement membrane and a thick stem extending to the lumen. Long processes issued laterally from its base, some lumenal and others with feet-like processes. Calcitonin gene-related peptide (CGRP) was present in about 20% of PNEC, mainly in the processes. CGRP-positive nerves were sparse, with some associated with the apical part of the PNEC.

**Conclusion:**

Our 3D-data demonstrates that PNEC are numerous and exhibit a heterogeneous peptide content suggesting an active and diverse PNEC population.

## Background

Pulmonary neuroendocrine cells (PNEC) are specialized airway epithelial cells that occur as solitary cells or as clusters called neuroepithelial bodies (NEB) [1]. They are located in the nasal respiratory epithelium, laryngeal mucosa [2] and throughout the entire respiratory tract from the trachea to the terminal airways [3]. In the fetal lung they are frequently located at the branching points of airway tubules, and in humans are present by 10 weeks gestation [4]. Neuroendocrine cells are bottle- or flask-like in shape, and reach from the basement membrane to the lumen. They can be distinguished by their profile of bioactive amines and peptides namely serotonin, calcitonin, calcitonin gene-related peptide (CGRP), chromogranin A, gastrin-releasing peptide (GRP) and cholecystokinin [4,5]. NEB may play a role as hypoxic-sensitive airway chemoreceptors [6], and an oxygen-sensitive potassium channel coupled to an oxygen sensory protein has been demonstrated in their lumenal membrane in the rabbit [7]. They are also considered to be involved in regulating localized epithelial cell growth and regeneration through a paracrine mechanism whereby their bioactive peptides are released into the environment [8]. Peptides and amines released by PNEC are involved in normal fetal lung development including branching morphogenesis [9]. The best-characterized peptides are GRP, the mammalian form of bombesin, and CGRP, which exert direct mitogenic effects on epithelial cells and exhibit many growth factor-like properties [10].

The majority of data available on the morphology, distribution, peptide expression and function of PNEC and NEB have been obtained from animal studies [11,12]. In human airways, the morphology of NEB have been studied ultrastructurally during the fetal and perinatal stage of lung development, and their peptides identified using immunogold-labeled antibodies where they are colocalized in the dense core vesicles in the cytoplasm [4,13-15]. However, there is little data describing the three dimensional morphology and peptide distribution in adult human airways where both PNEC and NEB are reported to be sparse [16,17]. It has been suggested that PNEC may play a role in mediating airway remodelling in normal lungs and in naturally occurring pulmonary disease where they increase in number [8,18].

The innervation of fetal and postnatal NEB has been also studied ultrastructurally in humans where both adrenergic and cholinergic nerve endings have been observed [4], in rabbits [19] and rats and dogs [20,21]. In rats, vagal nodose afferents traced using the carbocyanine dye DiI, terminate within NEB, but they are not positive for the sensory nerve marker CGRP [22] whereas the epithelium is richly innervated with CGRP- and Substance P (SP)- containing nerve terminals in guinea pigs [23], rats [22,24] and pigs [25]. In guinea pigs most of these afferents arise in the jugular ganglia [26,27]. However, little is known about the relationship between nerves and PNEC.

The aims of this study were to characterize the three dimensional morphology of PNEC, their distribution, and their relationship to the epithelial nerves in whole mounts of adult human bronchi using confocal microscopy. The peptides CGRP and GRP were examined for their consistency as markers of PNEC. Protein gene product 9.5 (PGP 9.5) was used as a marker of PNEC and for epithelial nerves, and CGRP for sensory nerves. The investigation was restricted to the solitary PNEC because NEB appear to be extremely rare in adult human lung [17].

## Methods

### Human airway tissue

A small section of bronchus was resected from the non-diseased portion of a lobe from a human lung obtained at thoracotomy from 8 patients undergoing surgery for the removal of lung tumors (Table 1). Three subjects were life-long non-smokers. The sample was removed from the freshly excised lobe on ice and fixed in Streck Tissue Fixative (Streck Laboratories, US). The airway segment(s) ranged from 3 to 6 mm inner diameter and were up to 1 cm in length. They were cut open lengthwise and the airway wall carefully dissected to create thin sheets that comprised epithelium and mucosa while discarding smooth muscle and cartilage. As a standard antigen retrieval method, tissues were microwaved for 20 min in citrate buffer (pH 6.0) and afterwards blocked for one hour in PBS pH 7.4 containing 1% bovine serum albumin. The tissue was cut into pieces of approximately 5 mm^2 ^area -usually about 10 pieces – which are referred to as wholemounts, and all further treatment was carried out in 96-well culture plates equipped with anti-evaporation lids.

**Table 1:** Patient Demographic Data

Patient	Gender	Age	Smoking status	Disease
1	Male	65	Smoker	SSC
2	Male	67	Smoker	LSC
3	Female	40	Non-smoker	Adeno
4	Female	37	Non-smoker	LSC
5	Female	61	Ex-Smoker	Adeno
6	Female	74	Smoker	SSC
7	Male	70	Smoker	SSC
8	Female	72	Smoker	SSC

Abbreviations: SSC, small cell carcinoma; LSC, Large cell carcinoma; Adeno, Adenocarcinoma.

### Preparation and staining of whole mounts

Whole mounts were stained with antibodies to reveal all nerves (PGP 9.5), sensory nerves (CGRP), and PNEC (PGP 9.5, CGRP and GRP). The PGP 9.5 antibodies (monoclonal and polyclonal) were obtained from UltraClone, UK and used at a dilution of 1/100 and 1/500, respectively. Antibodies to GRP (polyclonal) and CGRP (monoclonal and polyclonal) were purchased from Dako, NSW, Australia. The dilutions were as follows: GRP, 1/200; polyclonal CGRP, 1/400, monoclonal CGRP, 1/100. The secondary antibodies (anti-mouse and anti-rabbit) conjugated to Alexa-488 and Alexa-543, respectively were obtained from Molecular Probes, MA and used in a dilution of 1/200. Typically, 10 μl of antibody solution was used for each well. Control experiments to test for auto-fluorescence and non-specific staining were carried out using non-immune rabbit and mouse sera as described previously [28]. The tissues were incubated with primary antibodies overnight (4°C) in the presence of 0.3% Triton X-100 to enhance permeabilization. After washing 3 × 20 min in PBS, fluorophor-conjugated secondary antibodies were applied overnight (4°C). After further washing with PBS, the preparations were mounted in 90% glycerol containing p-phenylethylenediamine (1 mg/ml) to reduce bleaching of the fluorochromes. Custom-made slides were used that enabled imaging the specimen from both sides. The coverslips were raised with spacers (Imaging spacers, Sigma) in order to minimize compression of the specimens. The edges of the coverslips were sealed with nail polish to prevent evaporation of the mounting medium.

### Confocal microscopy

Wholemount pieces were double-stained in combinations of poly- and monoclonal antibodies and imaged using confocal microscopy (Biorad MRC-1000, Comos Software 7.0) as previously described [29]. For highest magnification, the focus depth was increased at 1 micron steps during scanning. The analysis and rendition of the resulting three-dimensional data sets, including side-projections, was performed using NIH-Image software (Version 1.61b12). Images were colorized and super-imposed with Adobe Photoshop 5 to reveal the complex structure of these cells. The Image Processing Tool Kit plug-in (Raindeer Software) was used to measure PNEC density. From each patient, a minimum of four fields of adequate staining quality and optimal signal to noise ratio, were imaged at a magnification of × 10. The PNEC were manually marked out and the software automatically calculated the PNEC density per area. Because the PNEC numbers were not homogeneously distributed in most of the bronchi sampled, no attempt was made to calculate the mean density of these cells for each bronchus.

## Results

### PNEC staining with PGP9.5

**Figure 1:**
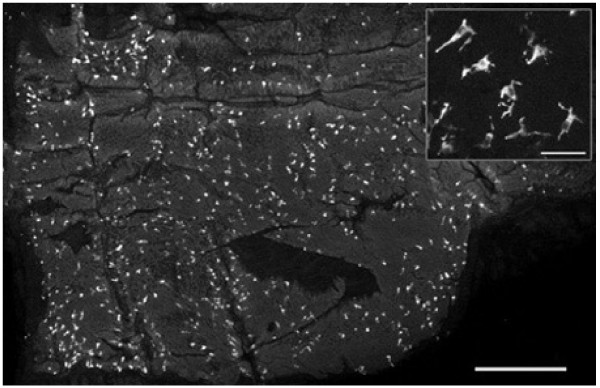
Whole mount of mucosa from a human bronchus stained with gastrin releasing peptide (GRP) and imaged from the luminal surface with a confocal microscope. The low power projection reveals an abundance of pulmonary neuroendocrine cells (PNEC) in the epithelium. Bar = 500 μm. Inset: higher power view revealing the morphology of PNEC. Where the epithelial surface is flat (ie. parallel with the cover slip), the view is from the top looking down on the cell body and processes but where they lie on the edge of a mucosal fold their flask-like shape is revealed. Bar = 50 μm.

**Figure 2:**
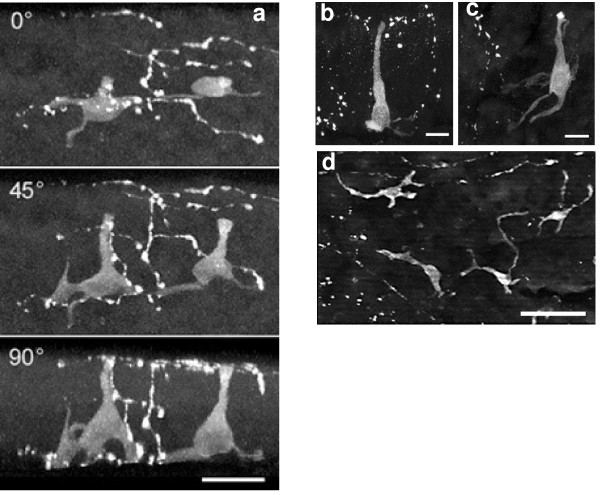
(a). High power projection of two PNEC and associated nerves imaged from the lumen and stained with the neural marker, protein gene product 9.5 (PGP 9.5). To reveal the shape of the cell body and the diverse structure of its processes the data set has been rotated to enable viewing the PNEC from the side. The upper panel is the conventional view from the lumen surface. The middle panel is rotated at 45 deg, and the lower panel shows the side view at 90 degrees. Nerves are present in close apposition to the PNEC. Bar = 20 μm. (b & c) Projections of typical flask-like PNEC stained with PGP 9.5 and imaged from the lumen over the edge of a mucosal fold. The cell bodies are seen from their sides (thus a cut off line for the surface of the epithelium with the lumen is not clearly seen). Processes of varying morphology arise from the cell bodies and varicose nerves are present near the base and apex of the PNEC with individual nerve fibers rising through the epithelium. The apex of the cell is brightly stained in (b). Bars = 10 μm. (d) Four PNEC viewed from a flat area of the airway lumen showing dendritic-like processes. This low power projection extends through a depth of 50 μm and includes nerves that lie below the basement membrane.

**Figure 3:**
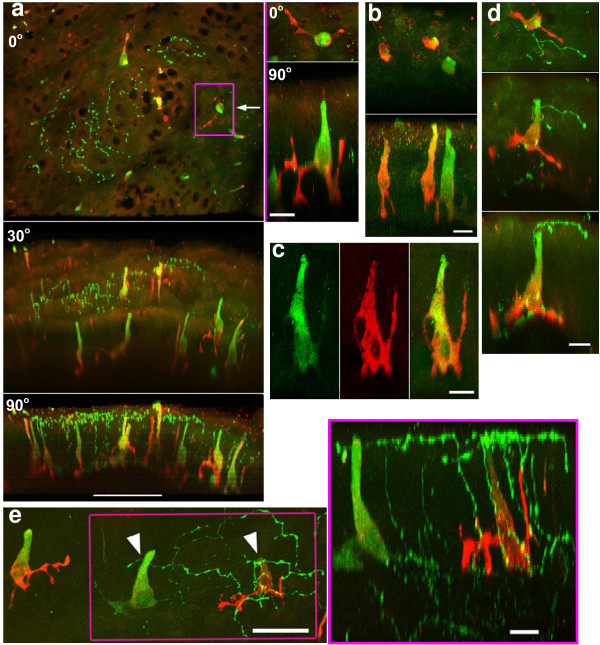
(a) Representative views of airway mucosa from four lungs double-stained for protein gene product 9.5 (PGP9.5, green) and gastrin releasing peptide (GRP, red). The upper panel is the lumen view, the middle one is rotated through 30 deg and the lower one through 90 deg, ie view from the side. Strings of varicose nerves are present in the epithelium. The dark holes indicate the location of goblet cells. PNEC are inconspicuous in the upper view but become more apparent when the field is projected at an angle. The lower panels demonstrate that GRP is present predominantly in the PNEC processes, whereas PGP 9.5 is restricted to the cell body with its apical stem. Nerves feature strongly in the apical epithelium. Bar = 50μm. *Boxed area*: This PNEC has been turned through 90 degrees so that the prominent processes now point upwards, and enlarged (right, upper panel). A 90 deg rotation of the projection reveals that the PNEC processes stain strongly for GRP whereas the cell body and apical stem stain mainly for PGP 9.5. Some of the processes are lumen-directed, other processes with feet-like appearance are directed toward the lamina propria. Bar = 10μm. (b) Three PNEC in a field from another lung shown from the lumen (upper) and rotated through 90 deg (lower). Two of the PNEC are predominantly GRP positive whereas the third PNEC stains strongly for PGP 9.5. The upper stem of the middle cell body stains yellow indicating that the PGP 9.5 and the GRP staining are about equal. Bar = 10 μm. (c) A single PNEC shown as individual fields: PGP9.5 only (left), GRP only (middle), composite PGP9.5 + GRP (right). GRP reveals fine processes that issue from the cell body. In contrast to PGP 9.5, GRP does not stain the cell nucleus. Bar = 10 μm. (d) A single PNEC in close association with a nerve terminal. A nerve rises from the base of the PNEC, climbs through the epithelium along the PNEC stem and spreads laterally in the apical epithelium where it exhibits enlarged terminal varicosities. Upper panel: lumen view, middle panel: 45 deg rotation, lower panel, 90 degree rotation. Bar 10 μm. (e) Lumen view of mucosa where the epithelium is tilted showing three PNEC from an angle. Patches of fine varicose nerves are present. Some of the nerves lie close to the stems of two of the PNEC (arrow heads). Bar = 25 μm. *Boxed area(right): *High power view after rotating shows two PNEC within a patch of nerves. The left PNEC is strongly PGP9.5 positive. The right PNEC has several processes in close apposition to nerves that rise through the epithelium to form an apical nerve plexus. Nerves in the apical epithelium lie close to the central stems of both PNEC. Bar = 10 μm.

Solitary PNEC were abundant within the epithelium of the bronchi of all eight human lungs examined when stained with PGP 9.5 or GRP. The numbers were not evenly distributed, ranging from 65 to 100/mm^2 ^over the area of any one wholemount, but with denser patches comprising 150 to 260/mm^2 ^observed less frequently in several of the lungs (Figure 1). Staining with the neural marker PGP 9.5 typically revealed PNEC with a flask-like shape when viewed from the side (Figure 2a, 90° rotation). The cell body was located near the basement membrane with the apical part of the cell comprising a characteristic thick stem that extended to the lumen surface (Figure 2b and 2c). The overall height of the cells averaged 50.1 ± 6.7 μm (SD, n = 21) in four lungs. Processes issued from the cell body along the basal region of the epithelium and also toward the lumen (Figure 2a, 90° rotation, Fig 2b and 2c). These processes have a dendritic-like appearance when viewed as a projection from the lumen because their three-dimensional morphology cannot be readily appreciated from this aspect (Figure 2a, 0° rotation; Figure 2d). Varicose nerves ascended in close association with the PNEC stem to reach an apical nerve plexus (Figure 2 and 3a, 3b) that lies just below the luminal surface of the epithelium. Figure 3a shows an example of an isolated patch of varicose epithelial nerves taken at low power (lumen view, 0 degrees). The apical disposition of these nerves is seen in the 90 degree rotation. No correlation was found between the distribution of PNEC and nerves in the epithelium.

### Co-localization of GRP and PGP9.5 in PNEC

All PGP 9.5-positive PNEC were also positive for GRP, however, the PGP 9.5 staining intensity of individual PNEC varied considerably. GRP exhibited significantly higher detail of the processes whereas PGP 9.5 stained predominantly the cell body with its prominent stem (Figure 3a, 30° and 90° rotations). This is strikingly shown in the PNEC enclosed in the boxed area of Figure 3a which has been rotated and enlarged. Most PNEC exhibited a dominance of one marker over the other (Figure 3a, 90 degree rotation, and Figure 3b). Individual staining of a single PNEC for both markers is shown in Figure 3c where detail of the thick and fine processes are revealed with GRP.

### Double staining for GRP and PGP9.5 in PNEC associated with nerves

The association of ascending and apical nerves with PNEC in the epithelium was more readily appreciated with double staining. Figure 3d shows a nerve rising from the base of a PNEC that extends upwards along the stem and terminates near the luminal surface. Figure 3e shows three PNEC, two of which are in a patch of nerves (arrows, boxed region). The right PNEC and its processes are in close proximity to a network of varicose nerves. When a rotation was performed on the field (boxed area, right) nerves arising from the base of this PNEC ascended along its stem to join the apical nerve plexus. Other nerves travelling from the lamina propria into the epithelium accompanied the GRP-stained processes towards the lumen.

### Double staining for CGRP and PGP 9.5

**Figure 4:**
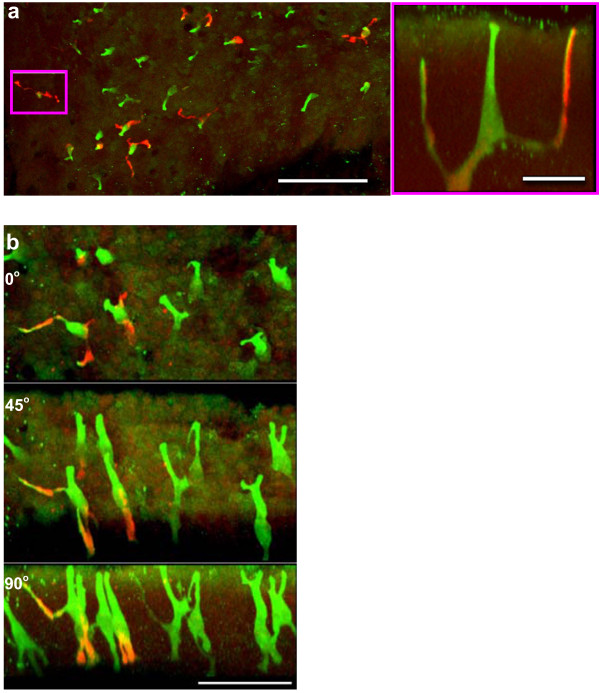
(a) Lumen view of a small area of epithelium representative of a whole mount of airway mucosa double-stained with calcitonin gene-related peptide (CGRP, red) and PGP9.5 (green). CGRP is present in terminal processes of some PNEC. Bar = 100 μm. *Boxed area (right): *Side projection of the PNEC where two lumen-directed processes and the long stem of the cell body (green) ascend to the apical epithelium. From this view the right process is strongly CGRP positive. Bar = 20 μm. (b) Wholemount of airway mucosa double-stained for CGRP (red) and PGP 9.5 (green) showing diverse morphology of PNEC. Upper panel is the lumen view, middle is a rotated through 45 degrees and the lower through 90 degrees. From left to right: A pair of PNEC with CGRP in the processes. The next two have fine processes that arise from the cell body and the apex of the stem. The right hand pair of PNEC exhibits branching of the main stem close to the lumen. Bar = 50 μm.

When PNEC were double stained for CGRP and PGP 9.5, CGRP was present in 22 ± 9% (SD, 10 fields, 4 lungs) of all PNEC stained (Figure 4a). CGRP typically stained the processes but was faint or absent in the cell body. Figure 4a (boxed area, right) shows a PNEC side-view with lumen-directed processes where one is particularly strongly stained for CGRP. It was also present in the shorter processes directed towards the lamina propria (Figure 4b, left) where three pairs of PNEC display quite diverse morphology. Unlike the staining pattern observed with GRP, CGRP is chiefly present in thicker, more proximal part of the processes.

### Staining for CGRP in nerves and PNEC

**Figure 5:**
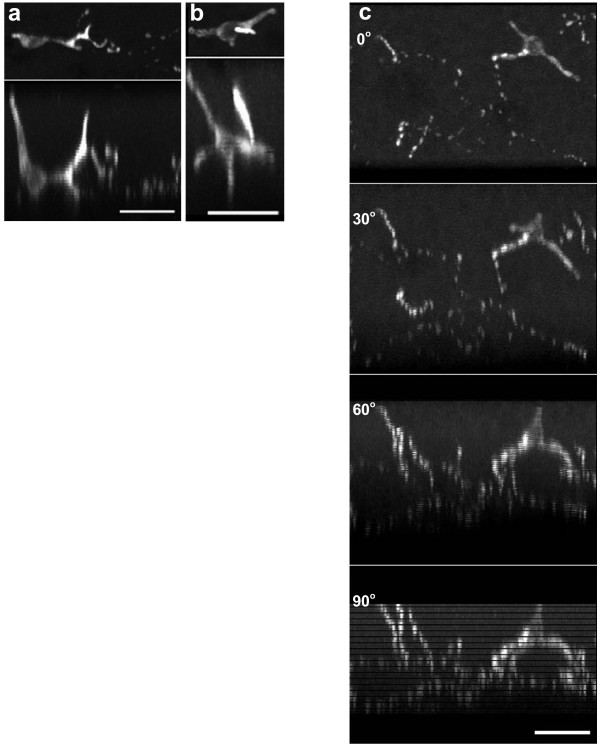
(a) Two PNEC stained with CGRP, shown as lumen view (upper panels) and rotated through 90 deg (lower panels). Each of the latter reveal a brightly staining process that issues from their cell body toward lumen whereas the cell bodies (left side of each panel) and other processes show weaker staining. Bar = 20 μm. (b) Wholemount of airway mucosa stained for CGRP. A network of weakly staining CGRP varicose nerves and a single PNEC is present. The top panel is the lumen view (0°), followed by rotations of 30°, 60° and 90°. One fiber exhibits brightly stained varicosities at the apparent point of contact with the PNEC. Rotating the field demonstrates that nerves run from below the base of the PNEC prior to ascending to it. Other ascending fibers are present in the left side of the field. The horizontal lines are a consequence of the limited number of Z steps in the confocal data set. Bar = 20 μm.

Faint staining of CGRP could be detected in PNEC cell bodies but it was much less than that in the processes. Figure 5a demonstrates that one of the lumen-directed processes stains stronger for CGRP than the other processes and the cell body. When PNEC were present in a field containing CGRP-positive nerves, they appeared to make contact with the PNEC. These contacts were characterized by brightly stained, enlarged terminal varicosities indicative of nerve endings, suggestive of innervation of PNEC by CGRP-positive nerves (Figure 5b).

## Discussion

This is the first report characterizing PNEC morphology in three dimensions in human airways. The use of whole mounts of mucosal tissue enabled the direct demonstration of PNEC abundance over large areas of airway epithelium. Rotation of 3-D images revealed the complexity of the PNEC body and its processes that issue laterally from its base, some lumen directed, others feet-like that were directed toward the lamina propria. PGP 9.5 and GRP were reliable markers, both staining all PNEC, whereas CGRP was present in about 20% of the PNEC population. However, the distribution of staining varied widely among cells, with GRP and CGRP mainly present in the processes, but with GRP occurring also in the cell body to varying degrees. The variation exhibited in the morphology of the PNEC and its differing peptide profiles suggests that these cells may be in a dynamic state in the epithelium.

PNEC were abundant when stained with either PGP 9.5 or GRP. Previous studies in human airways that investigated PNEC density used conventional cross sections and revealed numbers ranging from 1.05 PNEC/cm basement membrane (corresponding to 4 PNEC per 10,000 epithelial cells) using neuron specific enolase [17] to 12.5 PNEC/cm, or 41 PNEC per 10,000 epithelial cells, using chromogranin A as a marker [30]. In the current study, our measurements reveal that the density of PNECs ranges from 65/mm^2 ^to 260/mm^2 ^within an individual wholemount. There did not appear to be a homogenous distribution of PNEC either within or between wholemounts. Areas of high PNEC density appeared to be juxtaposed to areas of sparse cell numbers. Direct comparisons between the numbers provided in our study and those reported previously are confounded by the considerable discrepancy between the proportions of PNEC revealed by markers used to identify PNEC [17,30]. We used PGP 9.5 to label the whole PNEC population, whereas Boers reported that only 14% of all PNEC showed PGP 9.5 immunoreactivity [30]. Furthermore, our study indicates that all PNEC contain both PGP 9.5 and GRP, whereas Gosney et al., [17] found GRP present in 65% of all PNEC stained with neuron specific enolase and Boers demonstrated GRP in 59% of all PNEC stained with chromogranin A [30]. The reasons for these discrepancies are unknown, although the greater sensitivity and ability of confocal microscopy to resolve cell types and contents may account for the observed differences. Similarly, the size of the airway may also influence the PNEC distribution. In rat lungs at least, the density of NEB/PNEC appears to be dependent on airway size, with a greater density of cells observed in proximal airways compared to more distal generations [31]. Only large cartilaginous airways (3–6 mm ID) were available for this study. Although multiple wholemount pieces were analysed from each airway, our study was limited to 1 piece of bronchial tissue per subject and as such we are unable to comment on the overall distribution of PNEC throughout the lungs.

The majority of PNEC were not in close association with epithelial nerves, partly because nerves were generally sparse and tended to occur in patches as we reported recently [25]. When present, PGP 9.5 positive nerve fibres were generally observed to be in close apposition with the PNEC cell body, its stem, apex, and processes. Unfortunately, the immunofluorescent staining used in this study does not permit the distance between the nerve fibres and the PNEC to be measured accurately, and side projections computed from data obtained by scanning from the luminal surface are subject to loss of resolution. Nonetheless, when images were viewed from the lumen through a series of rotations over 360 degrees, it was clear that the nerves lie very close to these structures, within one micrometre. These are likely to be sensory nerves because they are varicose, with enlarged varicosities in the terminal region [25], and in most lungs stained positively though weakly for CGRP. CRRP-positive nerves endings appeared to terminate near or on the processes issuing from the base of the PNEC where bright terminal enlarged varicosities were seen. Afferent and efferent nerves have been characterized ultrastructurally on cells within NEB in fetal and neonate humans [4] and adult animals [21,22] but as far as we are aware, not on single PNEC. This approach is hampered because of the apparent paucity of epithelial sensory nerves in humans. The infrequent patches of PGP 9.5-positive epithelial nerves stain very weakly in humans [25,32], or not at all [33,34] for SP or CGRP [35] in contrast to rats [24] and pigs [25] where they are abundant.

In humans, NEB decrease in frequency with age and are rare in adult lung. Gosney et al.,[16] observed only three NEB after searching through preparations from 5 post-mortem specimens. Our data support this finding, as only two NEB were observed and were confined to a single lung after scanning several hundred whole mount PIECES from eight adult lungs. Brouns and colleagues recently demonstrated a complex innervation pattern of pulmonary NEBs in rat airways, comprising both sensory vagal nerves as well as non-vagal CGRP/SP positive nerves [36]. The physiological role of the innervation of NEB is not well understood. It has been proposed that the nerve endings at the base of the NEB subserve an axon reflex, presumably arising in the NEB itself and possibly penetrating to deeper tissues such as the airway smooth muscle [4]. There may also be local reflex connections through peripheral ganglia. Hypoxia detected by the O_2 _sensor in the NEB is presumed to release mediators that stimulate vagal afferents, but no central nervous reflexes have been identified [37]. Recent advances in microscopic techniques with increased sensitivity may shed more light on the morphological basis for many of the suggested functions of NEB innervation.

In our study, we used immunofluorescently-labeled antibodies to PGP 9.5, GRP and CGRP to detect PNEC in adult airway epithelium. PGP 9.5 stained all the cell bodies fairly uniformly but was weaker in the processes so that their fine ends frequently were not revealed. All PGP 9.5 positive cells were also positive for GRP, suggesting that it may also be a reliable marker for identifying PNEC. It was predominantly observed in the processes, but in many cells it stained the cell body and stem region either partially or completely. However, less than a quarter of PNEC exhibited CGRP immunoreactivity, with the greatest intensity displayed in the thick processes. Although CGRP is often used in animal studies as a marker for quantitative studies of NEB and PNEC [38], we have shown that CGRP is not a reliable marker of the PNEC populations in human adult epithelium, as only a subset exhibited CGRP immunoreactivity. These markers, used in conjunction with three dimensional imaging and image rotation, have revealed the overall morphology of the PNEC that hitherto has not been appreciated using conventional light and electron microscopy. PGP 9.5 revealed the variety of shapes that the cell body can attain, most often flask or bottle-shaped with the base at the basement membrane and its long stem extending to the lumen where its tip was often more brightly stained. Some PNEC exhibited branching of the main stem close to the lumen.

GRP staining revealed a striking PNEC morphology with thick processes issuing laterally from near the base of the cell body upwards toward the apical epithelium and along the basement membrane. In addition GRP also stained fine processes originating from the side of the PNEC body that were not readily detected with PGP 9.5. The thick and thin processes of the PNEC, revealed by our 3-D confocal microscopy, may be the conduits that effect delivery of the bioamines and peptides proposed to be secreted by PNEC.

Bioamines and peptides contained within the PNEC have been proposed to be secreted into the adjacent epithelium and lamina propria in response to such stimuli as hypoxia [6,11]. GRP and CGRP have been shown to have mitogenic and growth factor like influences [39] and may have a direct influence on epithelial regeneration and an indirect one via local vasodilation of the adjacent bronchial vasculature. Our confocal microscopic study demonstrates PNEC with heterogeneous peptide content, suggesting an active and diverse PNEC population is present in adult human airway epithelium.

In this study, lung tissue samples were derived from a diverse group of patients ranging from 39 to 74 years of age that were undergoing thoracotomy for removal of lung tumors. The small number of patients precluded the correlation of our results to gender or smoking history. Thus it is difficult to determine to what extent data presented in this study represent the steady-state versus disease-specific remodeling of the airway epithelium.

## Conclusion

Our 3D-data demonstrates that PNEC are numerous and exhibit a heterogeneous peptide content suggesting an active and diverse PNEC population. Valuable insights into the biology of cells identified in this study may come from a better understanding of their abundance, morphology and innervation comparing normal lung tissue versus injured or diseased lungs.

## Authors' contributions

MW, EJH carried out the sample preparation and confocal microscopy and reviewed the manuscript. MPS, PJT and DAK conceived of the program, participated in the design and coordination of this study, and drafted the manuscript.
